# Superficial Temporal Artery Pseudoaneurysm Diagnosed by Point-of-Care Ultrasound

**DOI:** 10.5811/cpcem.2018.11.40958

**Published:** 2019-01-07

**Authors:** Samuel L. Burleson, Francesca N. Cirillo, Courtney B. Gibson, John P. Gullett, David C. Pigott

**Affiliations:** University of Alabama, Department of Emergency Medicine, Birmingham, Alabama

## CASE PRESENTATION

**Image 1:**
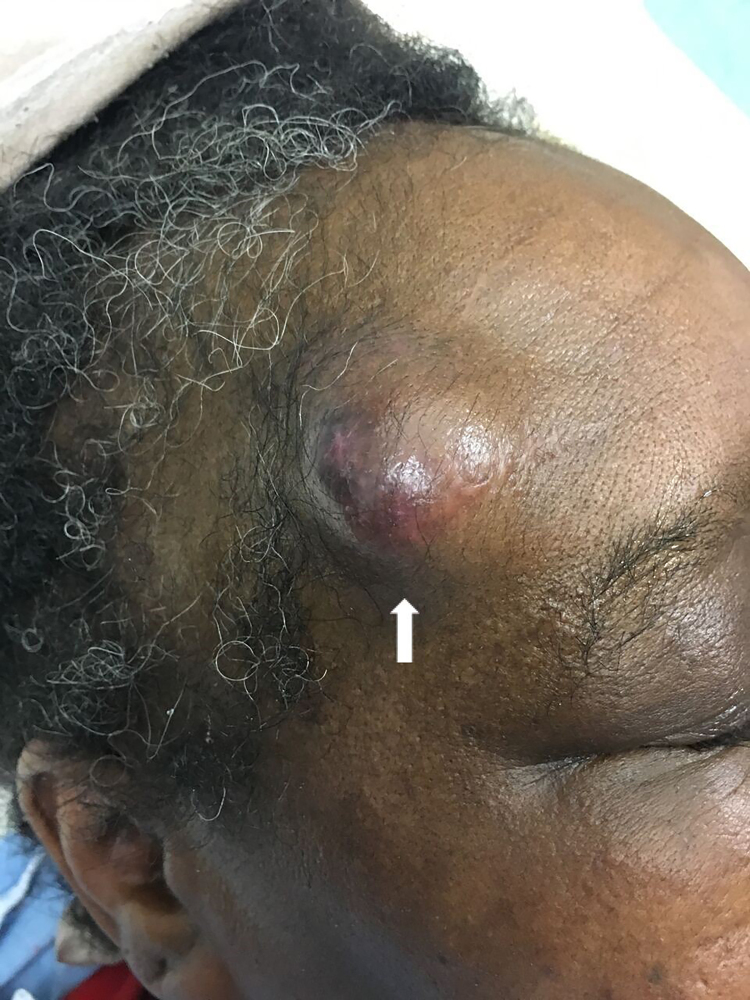
Photograph of right superficial temporal arterial pseudoaneurysm (arrow).

**Image 2:**
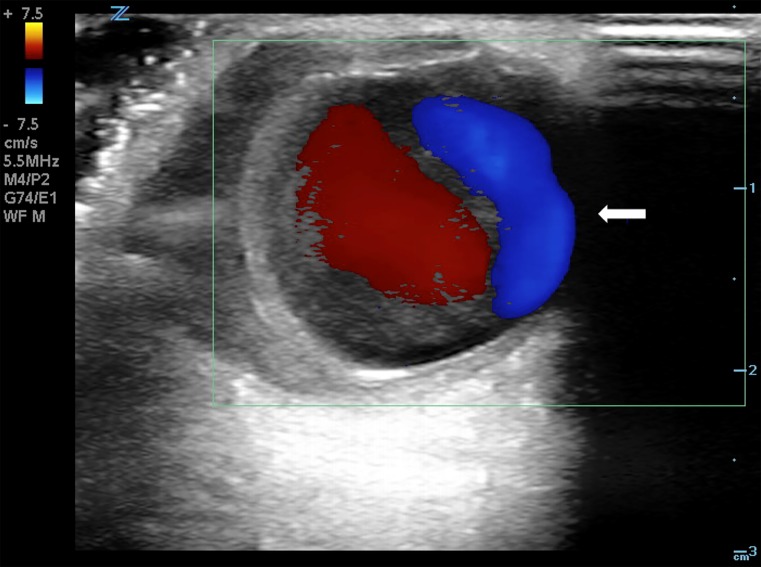
Point-of-care Doppler ultrasound demonstrating the classic “yin-yang” sign (arrow), indicating bidirectional blood flow within the pseudoaneurysm.

**Image 3:**
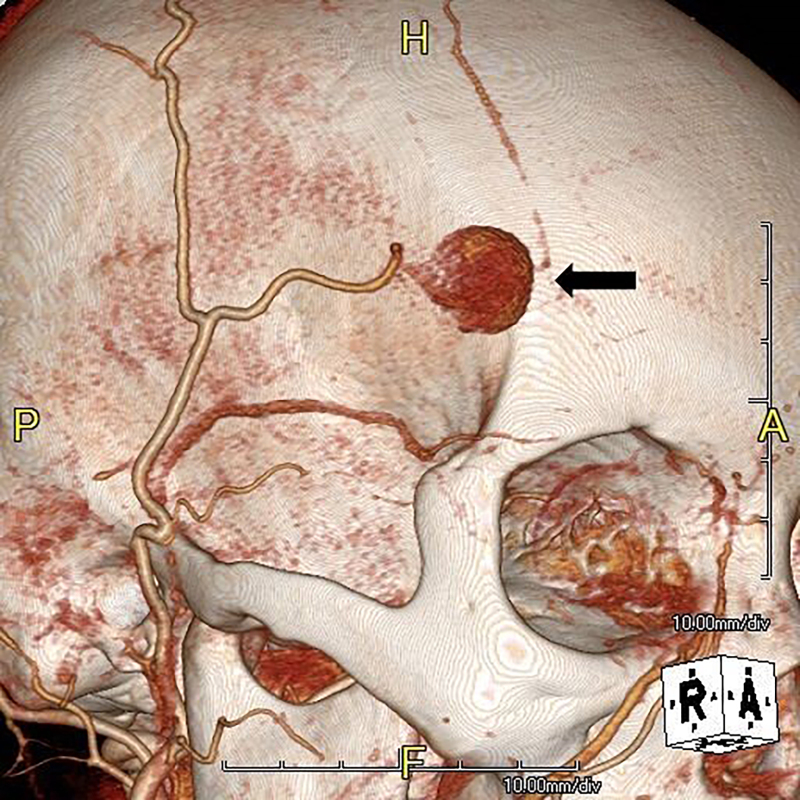
Three-dimensional computed tomography angiogram demonstrating a large superficial temporal arterial pseudoaneurysm (arrow).

A 55-year-old female presented to the emergency department with an enlarging forehead mass after a fall with head injury two weeks prior. She reported focal, tender swelling to her right forehead and headache. Physical examination revealed a two-centimeter, soft, pulsatile mass to her right frontotemporal region (Image 1). Point-of-care ultrasound (POCUS) with color Doppler revealed a dilated vascular structure with pulsatile, bidirectional flow – the “yin-yang” sign (Image 2). The diagnosis of traumatic superficial temporal artery (STA) pseudoaneurysm was confirmed by computed tomography (CT) angiography with three-dimensional reconstruction (Image 3). The patient’s pseudoaneurysm was surgically ligated and she recovered uneventfully.

## DISCUSSION

Traumatic pseudoaneurysm of the STA is a rare complication of minor head trauma, usually presenting as a painless pulsatile mass following blunt trauma.^1^ Pseudoaneurysms are contained only by the external adventitial layer of the vessel wall, and are more likely to rupture than true aneurysms.^2^ Complications may include persistent headache, continued enlargement, dizziness, vision changes and, rarely, life-threatening hemorrhage.^3^ Diagnosis is typically made by history and physical examination, and confirmed by Doppler ultrasonography^2^ or CT angiography, although diagnosis by POCUS has been reported.^2^,^4^,^5^

POCUS can differentiate common causes of focal, superficial swelling such as skin and soft tissue infection from underlying vascular pathology, preventing potentially disastrous attempts at bedside drainage and expediting referral for definitive therapy.^5^, ^6^ Color Doppler indicates blood flow velocity and direction relative to the probe, although high velocities may show an apparent reversal of color flow due to aliasing. The red-blue, “yin-yang” pattern seen here is due to the continuously changing angle of insonation caused by swirling blood flow.

In summary, we report a case in which POCUS provided a rapid, accurate diagnosis of an uncommon complication following minor head trauma requiring surgical intervention. An “ultrasound-first” approach to focal swelling, particularly with recent head trauma, can expedite appropriate care and avoid unnecessary or potentially harmful interventions.

**CPC-EM Capsule**

What do we already know about this clinical entity?

*Superficial temporal artery (STA) pseudoaneurysm is a rare complication of minor head trauma. Diagnosis is typically made via color Doppler ultrasound or computed tomography angiography*.

What is the major impact of the images?

*Point-of-care ultrasound (POCUS) with color Doppler assists in the diagnosis of STA pseudoaneurysm, as well as in undifferentiated soft tissue swelling*.

How might this improve emergency medicine practice?

*POCUS can expedite diagnosis and definitive therapy of STA pseudoaneurysm and help avoid harmful bedside interventions*.
